# Clinically Ambiguous Hemorrhagic Cardiac Tamponade Associated with Apixaban

**DOI:** 10.7759/cureus.24290

**Published:** 2022-04-19

**Authors:** Jose S Aguilar-Gallardo, Subrat Das, Pavan Reddy, Kiran Mahmood, Arieh Fox

**Affiliations:** 1 Internal Medicine, Icahn School of Medicine at Mount Sinai, New York, USA; 2 Mount Sinai Heart, Icahn School of Medicine at Mount Sinai, New York, USA

**Keywords:** acute cardiac care, initial presentation, direct oral anticoagulants (doac), cardiac tamponade, hemopericardium

## Abstract

Hemorrhagic cardiac tamponade in the setting of direct oral anticoagulants (DOACs) is rare but life-threatening. Presentation in subacute cases can also be nonspecific, which can potentially delay diagnosis. A 60-year-old female with a history of heart failure and chronic obstructive pulmonary disease presented with shortness of breath, chest pain, and cough while on treatment with apixaban after a recent hospitalization for pulmonary embolism. Clinical presentation was consistent with multiple diagnoses, including pneumonia and heart failure exacerbation. However, there were several risk factors for hemopericardium with DOACs such as elevated creatinine, hypertension, elevated international normalized ratio (INR), and concomitant use of medications with similar metabolic pathways as apixaban. In addition, subtle findings on examination such as oximetry paradoxus and electrical alternans were crucial for an early diagnosis and management. In this case, we discuss key characteristics of hemopericardium with DOACs, as well as considerations on its management.

## Introduction

Hemorrhagic cardiac tamponade is a life-threatening condition, so expedited diagnosis and management are essential, and early clinical suspicion can make a significant difference in patient outcomes. However, subacute cases can have a nonspecific presentation, and their infrequent nature can delay diagnosis. Pericardial bleeding with direct oral anticoagulants (DOACs) has been reported with an incidence of up to 0.05% in randomized controlled trials [1]. A systematic review reported 27 cases of hemorrhagic cardiac tamponade with DOACs, of which only six were with apixaban [2]. Signs and symptoms of subacute cardiac tamponade can be very nonspecific, varying from being asymptomatic to symptoms of shock [3]. Hemorrhagic cardiac tamponade secondary to DOACs has presented most commonly with shortness of breath (73%) and chest pain (38%) [2]. This case was presented as a poster at the American College of Cardiology 2022 Annual Scientific Session.

## Case presentation

A 60-year-old female presented with exertional shortness of breath and chest pain. She reported residual shortness of breath after a recent hospitalization for pulmonary embolism (PE) three weeks before, receiving treatment with apixaban 5 mg twice daily. She had a history of chronic diastolic heart failure, chronic obstructive pulmonary disease, systemic lupus erythematosus (SLE), chronic kidney disease (CKD), active tobacco use, anemia with baseline hemoglobin 7-8 g/dL, and type 2 diabetes mellitus. Symptoms were followed by one week of stabbing, non-exertional chest pain that radiated to the back, worse in the lateral decubitus position, and associated with fever and dry cough. Vital signs revealed tachycardia with a heart rate of 120, tachypnea with a respiratory rate of 30, fever of 38.4 ^o^C, and blood pressure of 157/74 mmHg. A physical examination revealed jugular venous distention (JVD) of about 12 cm. Cardiac auscultation revealed a regular rhythm without murmurs or rubs. Bibasilar lung crackles were appreciated. There was no lower extremity edema.

The patient’s presentation was consistent in at least two diagnoses, which results in hospital admissions. Pneumonia was suggested by respiratory symptoms with fever, tachycardia, and tachypnea. Heart failure exacerbation was suggested by exertional shortness of breath with volume overload and bilateral pleural effusions. There was a moderate risk of PE given tachycardia, and recent PE also suggested a persistent thrombus as contributing to symptoms. The pericardial disease was suggested by positional chest pain, JVD, and fever.

**Figure 1:**
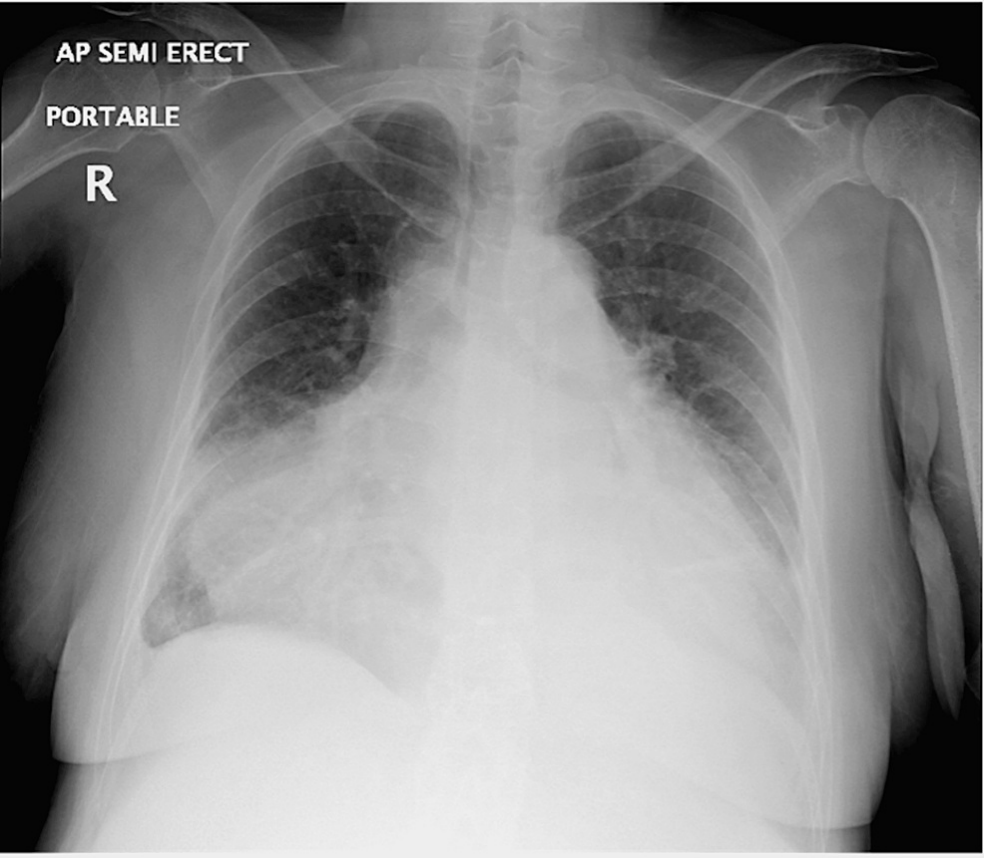
Chest X-ray. Enlarged cardiac silhouette corresponding to a “water bottle sign”

**Figure 2:**
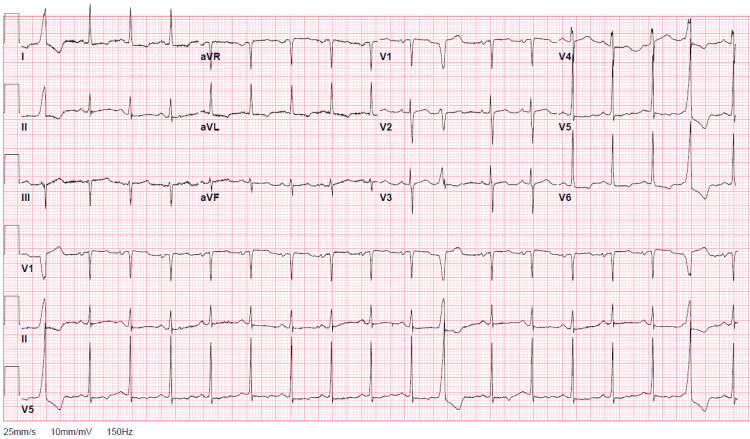
Electrocardiogram. Subtle “electrical alternans”

Laboratory findings included anemia with a hemoglobin count of 7.2 g/dL, a normal white cell count of 10.50$\times$10^3^ μl, an elevated serum creatinine value of 1.46 mg/dL, normal blood urea nitrogen content of 19 mg/dL, an elevated international normalized ratio (INR) of 1.4, elevated brain natriuretic peptide of 644 pg/mL, normal troponin of 0.02 ng/mL, elevated D-dimer of 14.48 µg/ml fibrinogen equivalent units (FEUs), and elevated C-reactive protein (CRP) of 146 mg/L. SARS-CoV-2 molecular assay was negative. Chest X-ray was notable for a markedly enlarged cardiac silhouette as well as small bilateral pleural effusions with possible subadjacent consolidation (Figure 1). The electrocardiogram was notable for sinus tachycardia with premature ventricular contractions, lateral T-wave inversions, and subtle variation in amplitudes between QRS complexes consistent with “electrical alternans” (Figure 2).

**Figure 3:**
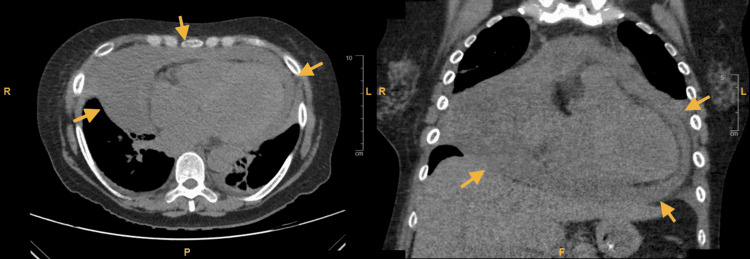
Chest CT. Large hemopericardium (arrows) CT, computed tomography.

The patient initially received ceftriaxone and azithromycin for community-acquired pneumonia, as well as intravenous furosemide. She was continued on home dose hydroxychloroquine and received one dose of home apixaban. Given the history of nonspecific, persistent respiratory symptoms, a CT scan of the chest was taken, revealing a large pericardial effusion with a radiodensity of 20-30 Hounsfield units, suggestive of hemopericardium (Figure 3). There was no evidence of right heart strain or focal consolidation. Transthoracic echocardiography found a large pericardial effusion without signs of chamber compression, dilated inferior vena cava, dilated left atrium, and left ventricular hypertrophy with an estimated ejection fraction of 50% (Video 1). Clinical examination with a manual blood pressure cuff revealed pulsus paradoxus, quantified at 25 mmHg. Examination of pulse oximetry tracing revealed “oximetry paradoxus.”

**Video 1:** Transthoracic echocardiogram. Large pericardial effusion TTE, transthoracic echocardiogram.

After diagnosing hemorrhagic tamponade, apixaban was discontinued, and the patient was started on colchicine. Immediate pericardiocentesis drained 400 cc of the bloody fluid. Fluid microscopy revealed blood and no malignant cells. Subsequent drainage amounted to 200-400 cc daily, for which a pericardial window was performed after five days. A sample of pericardial tissue was sent for biopsy. Subsequent lower extremity ultrasound revealed an acute deep venous thrombosis, prompting placement of an infrarenal inferior vena cava filter. CT angiography of the chest was negative for PE.

Pericardial drainage receded (Video 2), and the drain was removed after an additional four days. The patient continued treatment with colchicine.

**Video 2:** Transthoracic echocardiogram after drainage of pericardial effusion. Improvement in effusion is seen TTE, transthoracic echocardiogram.

**Figure 4:**
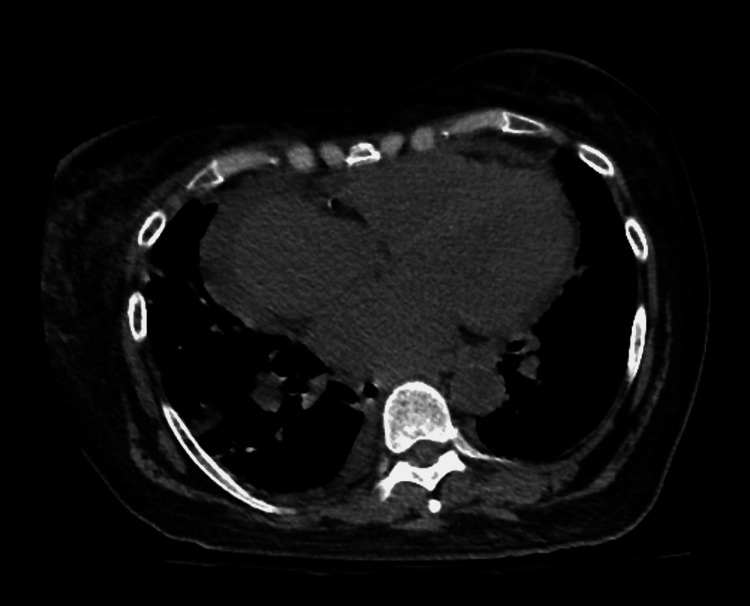
Chest CT 3 weeks before patient presentation. Cardiomegaly with no pericardial effusion CT, computed tomography.

Subsequently, a diagnosis of exclusion attributed the pericardial hemorrhagic to the initiation of apixaban. Malignancy was ruled out with negative fluid cytopathology, which revealed red blood cells in an otherwise acellular fluid, as well as negative pericardial biopsy results including cytokeratin immunostains, which revealed mesothelial cells and fibrinous material with inflamed pericardial tissue. Tuberculous pericarditis was ruled out with a negative interferon-gamma release assay, and other bacterial etiologies were less likely given negative gram stain and cultures and a lack of inflammatory cells in pericardial fluid. Persistently elevated CRP raised suspicion for SLE pericarditis; however, autoimmune serology was negative including anti-double-stranded DNA, anti-Smith, ribonucleoprotein antibodies, and beta 2 glycoprotein with normal complements C3 and C4, and no other clinical findings suggestive of a lupus flare. Uremic pericarditis was ruled out with a normal blood urea nitrogen with stable kidney disease not needing dialysis. Finally, a review of chest CT (Figure 4) and echocardiography of recent admission three weeks before was negative for pericardial effusion, establishing a temporal connection with the initiation of apixaban.

The patient’s clinical condition improved, with eventual discharge home. Upon follow-up, she reported complete resolution of symptoms.

## Discussion

Analysis of risk factors raises suspicion of DOAC-associated hemopericardium. Based on reported cases, the patient had multiple risk factors that include older age (>65 years), hypertension, elevated creatinine, and elevated INR [2]. The patient was also on multiple drugs metabolized by cytochrome P450 3A4 (amlodipine, losartan, escitalopram, and hydroxychloroquine), which could lead to increased apixaban exposure [4]. Of note, while elevated creatinine is a reported risk factor, apixaban has been used effectively in CKD. Full-dose apixaban has been seen to improve outcomes when compared to warfarin in the context of atrial fibrillation and advanced CKD, and guidelines recommend its use in end-stage kidney disease [5].

Physical examination maneuvers specific to cardiac tamponade can be key to clinical diagnosis. Pulsus paradoxus greater than 10 mmHg in a patient with a pericardial effusion increases the likelihood of tamponade with a likelihood ratio (LR) of 3.3 (95% CI, 1.8-6.3), while if greater than 12 mmHg, then the LR increases to 5.9 [3]. A study also found that an “oximetry paradoxus” ratio (the ratio from maximum to minimum amplitude in the recorded pulse oximetry waveforms) of ≥1.5 had a sensitivity of 80% and specificity of 81% for tamponade [6]. Routine imaging can also provide clues, such as a “water bottle sign” on a chest X-ray and “electrical alternans” on an electrocardiogram.

Serum monitoring with a direct thrombin inhibitor and anti-Factor Xa assays has shown promising utility and can be considered in patients with a high risk of bleeding, which requires more research [7]. Furthermore, the prediction of drug-drug interactions with apixaban could be useful to prevent bleeding, especially in patients on multiple medications. Artificial intelligence and neural network-based methods have been seen to be effective at predicting interactions [8].

Discontinuation of apixaban should occur promptly upon signs of bleeding. Packed red blood cells should be transfused as necessary based on hemoglobin levels, and four-factor prothrombin complex concentrate should be administered if bleeding is deemed life-threatening or uncontrolled [9]. Andexanet alfa has also been approved by the U.S. Food and Drug Administration for the reversal of apixaban in certain scenarios [9]. In cardiac tamponade, guidelines recommend immediate pericardiocentesis, which can have hemodynamic benefits even in smaller pericardial effusions that have intrapericardial pressures that are lower than right atrial pressures [10,11]. Such benefits involve a decrease in ventricular filling pressures and the inspiratory reduction of arterial systolic pressure [11]. Other indications include symptomatic moderate or large pericardial effusions not responsive to medical therapy or with an unknown associated disease [10]. A pericardial window is indicated for recurrent large pericardial effusions [10].

## Conclusions

To conclude, hemorrhagic cardiac tamponade secondary to apixaban is rare and can be missed on initial assessment. Availability bias and an ambiguous presentation can hamper early clinical suspicion. Analysis of risk factors and diagnosis-specific examination maneuvers can be key to a timely diagnosis and management.
